# Efficacy of *Trichoderma longibrachiatum* Trichogin GA IV Peptaibol analogs against the Black Rot Pathogen *Xanthomonas campestris* pv. *campestris* and other Phytopathogenic Bacteria

**DOI:** 10.3390/microorganisms11020480

**Published:** 2023-02-14

**Authors:** Rocco Caracciolo, Luca Sella, Marta De Zotti, Angela Bolzonello, Marco Armellin, Livio Trainotti, Francesco Favaron, Silvio Tundo

**Affiliations:** 1 Department of Land, Environment, Agriculture and Forestry (TESAF), University of Padova, 35020 Legnaro, Italy; 2 Department of Chemistry, University of Padova, 35131 Padova, Italy; 3 Department of Biology (DiBio), University of Padova, 35121 Padova, Italy

**Keywords:** gram-negative bacteria, antibacterial activity, seedborne pathogen, cytotoxicity, cauliflower, antimicrobial peptides (AMPs)

## Abstract

Black rot caused by the Gram-negative bacterial pathogen *Xanthomonas campestris* pv. *campestris* (Xcc) is considered one of the most destructive diseases affecting crucifers. Xcc is a seedborne pathogen able to infect the host at any growth stage. The management of the pathogen mainly relies on the use of copper-based products with possible negative effects on human health and the environment. Searching for protection alternatives is crucial for achieving a sustainable management of Xcc. *Trichoderma* spp. has been largely used as a biocontrol agent against several phytopathogens. Among *Trichoderma* species, *Trichoderma longibrachiatum* produces the peptaibol trichogin GA IV, a secondary metabolite with antimicrobial activity against Gram-positive bacteria, as well as filamentous and yeast-like fungi. In this work, we tested, at micromolar concentrations, 25 synthetic analogs of the peptaibol trichogin GA IV for their bacteriostatic and bactericidal activity toward the bacterium Xcc. One of the most effective peptides (4r) was also tested against the Gram-negative bacteria *Xanthomonas arboricola*, *Pseudomonas corrugata*, *Pseudomonas savastanoi* pv. *savastanoi*, *Agrobacterium tumefaciens*, *Ralstonia solanacearum*, and *Erwinia carotovora* subsp. *carotovora*, as well as the Gram-positive bacterium *Bacillus subtilis*. The peptide 4r reduced black rot symptoms on cauliflower plants when administered both before and 24 h after inoculation with Xcc. The cytotoxic activity of the peptide 4r was also evaluated towards suspensions of tobacco cells by Evans Blue assay.

## 1. Introduction

Plant diseases caused by bacterial pathogens represent major constraints on crop production and cause severe losses in agricultural yield worldwide [1]. Among bacterial phytopathogens, the Gram-negative *Xanthomonas campestris* pv. *campestris* (Xcc) is of particular interest because of its wide host range. Xcc is listed in the ‘Top 10′ list of the most important bacterial plant pathogens [2]. Indeed, it is pathogenic to several *Brassica* species, including economically important crops such as cabbage, on which the pathogen causes black rot disease [3]. Plants belonging to the Brassicaceae family are susceptible to the disease in all developmental stages. The symptoms are characterized by V-shaped lesions affecting the leaf margins. Veins of infected leaves, stems and roots turn black as the bacterium enters the vascular tissues. Notably, PCR-based assays for the early detection of Xcc have been developed [4]. Since the use of plant host resistance is hampered by the scarcity of available resistant varieties, management strategies are limited to the use of certified pathogen-free seeds and transplants, cultural practices aimed at inoculum reduction, and chemical control with copper-based products [3]. Reliance on copper-based products will suffer the effects of the new regulatory requirements, which were implemented based on human health and environmental concerns. Indeed, the European Union (EU) has implemented regulatory plans to reduce the application of copper (regulation EC 2018/1981) for plant protection. Biocontrol agents (BCAs) belonging to *Bacillus* and *Pseudomonas* spp. and bio-based extracts showed antagonistic activity toward the pathogen [5,6] and reduced black rot severity in kale plants, respectively [7]. However, BCAs could have limited effectiveness against Xcc in the open field, related to variable weather conditions, the local environment and formulation. Due to these drawbacks, the effective control of Xcc with BCAs still represents a challenge. Therefore, the search for new alternative strategies that are effective against bacterial pathogens is highly required for sustainable plant disease management. In this regard, the adoption of antimicrobial peptides (AMPs) is receiving increased attention [8]. AMPs are non-toxic and eco-friendly bioactive molecules that are produced by diverse organisms, such as fungi, bacteria, animals, and plants [9]. An interesting feature of these molecules is the antimicrobial activity exerted against pathogenic fungi, yeasts, insects [9] and, interestingly, bacteria belonging to the genera *Pseudomonas* and *Xanthomonas* [10,11]. Recently, different AMPs used as individual molecules or in mixtures showed bioactivity against the bacterial pathogen *Erwinia amylovora* [12,13], suggesting their use as a control measure against this bacterium. The increasing interest in AMPs as a new management strategy for bacterial diseases is also related to their low propensity to induce resistance in bacteria [14].

Among the organisms identified as AMP producers, the biocontrol agent *Trichoderma* spp. has been described as a source of bioactive molecules for controlling phytopathogens [15]. The antagonistic capacity of *Trichoderma* spp. is also exerted through the release of a specific class of AMPs called “peptaibols”, characterized by the presence of several natural non-coded α-aminoisobutyric acid (Aib) residues, an acyl group at their N-terminus and a 1,2-aminoalcohol at their C-terminus. Peptaibols exert their antimicrobial activities mainly through a perturbation of the permeabilization of the pathogen membrane [16]. The present study focused on the *Trichoderma longibrachiatum* short-length peptaibol trichogin GA IV [17,18]. Its sequence features an *n*-octanoyl (n-Oct) at the N-terminus and the 1,2-aminoalcohol leucinol (Lol) at the C-terminal end [19]. In our previous study, we designed several analogs of trichogin GA IV, with Gly to Lys substitutions, to increase its hydrophilicity and antimicrobial activity [20]. Some of these analogs inhibited *Botrytis cinerea* growth in vitro and significantly reduced gray mold symptoms on leaves of common bean, grapevine and tomato, and on ripe berries of grapes [20,21]. The antimicrobial activity was also demonstrated against the hemibiotrophic fungal pathogen *Pyricularia oryzae* [22]. Interestingly, trichogin GA IV is reported as having antimicrobial activity against Gram-positive and not against Gram-negative bacteria [23]. In this work, we established the bactericidal activity of derived trichogin analogs [20] against the plant pathogenic bacterium Xcc in vitro and verified the possibility to control black rot in cauliflower plantlets. Besides, one of the most effective peptides against Xcc was also assayed against several other pathogenic bacteria, namely the Gram-negative bacteria *Xanthomonas arboricola*, *Pseudomonas corrugata*, *Pseudomonas savastanoi* pv. *savastanoi*, *Agrobacterium tumefaciens*, *Ralstonia solanacearum*, *Erwinia carotovora* subsp. *carotovora* and the Gram-positive BCA bacterium *Bacillus subtilis.*

## 2. Materials and Methods

### 2.1. Trichogin GA IV Synthetic Analogs Used in This Work

Trichogin GA IV analogs were synthesized as previously described [20]. Lyophilized peptides were solubilized in sterile Milli-Q water to a final concentration of 1 mM. A stock solution of 100 µM was prepared for each peptaibol, in order to perform both in vitro and in vivo assays. Suspensions were then stored at −20 °C. Sequences of the tricogin GA IV analogs used in this work are reported in Table 1. C-terminal-modified analogs carrying a C-terminal amide (2r, 4r, 5r, 6r, 22r and 24r) and three short versions of peptide 4 were also synthesized (4c, 4c1 and 4c2).

### 2.2. Xcc Isolation and Identification

The *X. campestris* pv. *campestris* strain RC1 used in this work was isolated as reported in [24], from symptomatic cauliflower leaves sampled from a field in the Veneto Region in the municipality of Padova. The identification of the bacterium was carried out by sequencing the *16S* region [25] and the *hrpF* gene [26] with the primer pairs 27F (5′-AGAGTTTGATCCTGGCTCAG-3′)—1492R (5′-GGTTACCTTGTTACGACTT-3′) and XCF (5′-CGATTCGGCCATGAATGACT-3′)—XCR 5′-CTGTTGATGGTGGTCTGCAA-3′, respectively.

### 2.3. Antimicrobial Activity of Peptaibols

The antimicrobial activity of the 25 peptaibol analogs of trichogin GA IV against Xcc was evaluated through the following growth inhibition assay. Twenty microliters of Xcc bacterial suspension at a concentration of 10^6^ cfu/mL was mixed with 100 µL of Luria Bertani Broth (LB) 2X. Then, aliquots of each peptide were added to obtain concentrations of 15 and 3 µM, and the solution was brought to a final volume of 200 µL with sterile deionized water. Three replicates were performed for each peptide dose. The plates were incubated at 27 °C ± 1 for 72 h. Bacterial growth was determined by measuring optical density at 600 nm with a Multiskan™ FC Microplate Photometer (Thermo Scientific™, Waltham, MA, USA), after 20 s of gentle suspension agitation [10,27].

### 2.4. MIC and MBC Determinations

The Minimal Inhibitory Concentration (MIC) of the most effective peptides against Xcc was measured through the growth inhibition assay, as described before. The MIC was defined as the lowest concentration of peptide that completely inhibited visible growth. Peptaibols were tested at decreasing concentrations (3, 2, 1 and 0.5 µM). Minimal Bactericidal Concentration (MBC) was determined at the end of the incubation period (72 h) by plating, onto LBA plates, 5 µL samples that were collected from each well with no visible growth. The MBC was defined as the lowest concentration of peptides that did not allow bacterial growth of the pathogen on the plate after 24 h.

### 2.5. Peptide 4r Activity against Gram-Negative Bacterial Plant Pathogens and the Gram-Positive Bacterium B. subtilis

The activity of the 4r analog was evaluated by the in vitro antimicrobial activity assay described above against several Gram-negative plant pathogens, namely *P. corrugata* strain 2445, *A. tumefaciens* strain 2437 and *E. carotovora* subsp. *carotovora* strain 2577, derived from the National Collection of Plant Pathogenic Bacteria (NCPPB), *X. arboricola* strain 191, *P. savastanoi* pv. *savastanoi* strain 326, and *R. solanacearum* strain 127, derived from the collection of bacterial pathogens of the University of Tuscia (Department of Agriculture and Forest Sciences), as well as the Gram-positive bacterium *B. subtilis* strain QST 713, which is the active ingredient of Serenade Aso (Bayer Crop Science, Leverkusen, Germany).

The 4r peptide was tested at the concentrations of 3 µM and 15 µM; three biological replicates were performed for each bacterium. Two independent experiments were conducted.

### 2.6. Tobacco Cell Culture Maintenance and Evans Blue Assay

*Nicotiana tabacum* cv. Samsung NN cell cultures were maintained in 10 mL liquid medium (4.4 g/L sucrose, 1×Murashige Skoog Salt Mixture and Vitamins, 0.5 mg/L 2,4-dichlorophenoxyacetic acid, 0.25 mg/L 6 Benzylaminopurine, pH of 5.5 adjusted with KOH) in a 50 mL flask, with orbital shaking (100 rpm) in the dark at 25 °C; these cultures were sub-cultured every week. The protocol for cell culture maintenance was adapted from [28]. Cytotoxicity was determined on a one-week cell culture suspension after incubation for 12 h with the peptide 4r [29] at different concentrations (3, 15, 30 and 50 µM). As a negative control, the cells were treated with BSA or water. As a positive control, the cells were killed by heating them in a boiling bath for 5 min. A 1 mL cell suspension was stained with 0.025% (*v/v*) Evans blue for 15 min at RT. The cells were then extensively washed with water and suspended in 50% (*v/v*) methanol with 1% (*w/v*) SDS at 60 °C for 30 min. Afterward, the cells were centrifuged at 2000× *g* and the supernatant was diluted to a final volume of 7 mL. Absorbance was quantified in microtiter plates filled with 200 µL of the diluted supernatant at 595 nm, by using a Multiskan FC Microplate Photometer (Thermo Scientific). Three technical and three biological replicates were used in three different experiments.

### 2.7. Xcc Inoculation Assays

Seeds of white cauliflower cv. “Palla di neve” were placed in a Petri dish containing filter paper soaked with 2 mL of sterilized distilled water (SDW). The dish was sealed with parafilm and incubated at 25 °C for 48 h. Sprouts were planted in 6-cell peat trays (4 × 4 × 6 cm) containing mixed peat organic substrate, and placed in a controlled environment at 22 °C with a 16 h photoperiod and 55% of RH. After 15 days, seedlings were transferred to single plastic pots (7.5 × 7.5 × 6 cm).

Treatments and foliar inoculations with a bacterial suspension were performed two weeks after repotting. According to the experimental plan, the following treatments were compared: the peptide 4r at 50 µM in presence of 2.5% (*v/v*) of the tackifier pinolene (Nu-Film-P, BIOGARD, Grassobbio, Italy), the pinolene solution at 2.5%, the cupric commercial bactericide Tricopperland (tribasic copper sulfate, ISAGRO s.p.a., Milano, Italy) at a concentration of 1.6 g/L (0.48 g/L of copper metal), and deionized water.

The treatments were performed by spraying before (pre-inoculation) or after (post-inoculation) inoculation with Xcc. In the pre-inoculation experiments, 5 mL of each preparation was sprayed on the adaxial surface of the leaves and allowed to dry. Then, a bacterial suspension grown for 24 h containing about 10^8^ cfu/mL was mixed with 2.5% pinolene, drawn with a 1 mL syringe, and inoculated onto the treated leaf by scratching the surface of the midrib with the dripping tip of the needle. To favor infection, the inoculated seedlings were enveloped in moistened bags for 24 h.

In the post-inoculation experiments, leaf sprays with bactericidal preparations were performed 24 h after the bacterium inoculation, performed as reported above, once the bags were removed.

For each treatment, 2 leaves of 3 plants were inoculated. Three different treatments were applied before and after inoculation with Xcc. Three independent experiments were performed (9 plants and 18 leaves per treatment).

Ten days post-inoculation (dpi), leaves were collected and scanned at 600 dpi, and the resulting images were used to analyze symptomatic and total leaf areas by Assess 2.0©: Image Analysis Software for Plant Disease Quantification (American Phytopathological Society, St. Paul, MN, USA). These values were used to determine the disease severity area (DSA) percentage as follows: DSA (%) = (area_lesion_/area_leaf_) × 100.

### 2.8. Statistical Analysis

In vitro assays were statistically analyzed by one-way ANOVA followed by the Tukey–Kramer Post Hoc Test at *p* < 0.05. Infection results were statistically analyzed by R software version 4.2.2 [30]. Data were submitted to an analysis of variance according to a linear model, considering the treatments as a fixed effect, and the averages were separated by Tukey’s honestly significant differences tests. Non-normal data distributions were normalized with the function log (x). To assess the goodness-of-fit of the model, the normality of residuals was also tested.

## 3. Results

### 3.1. Antimicrobial Activity of Peptides against Xcc

**Figure 1:**
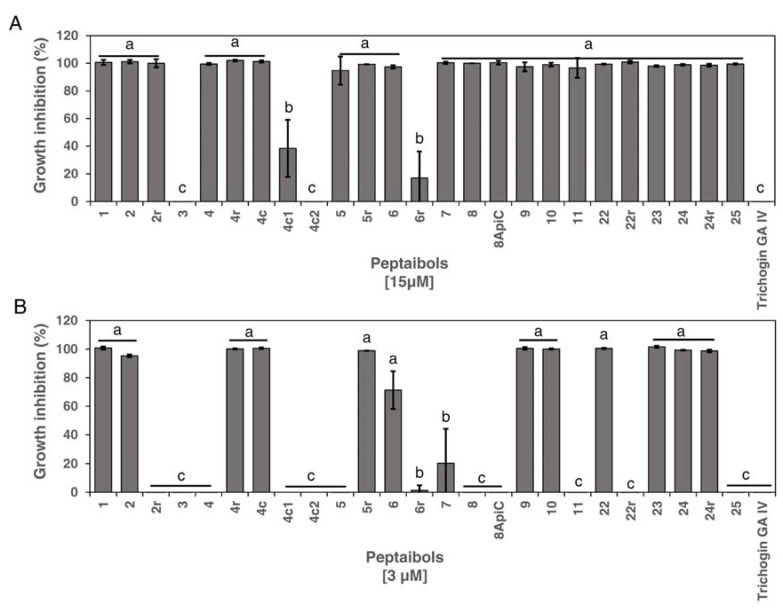
Efficacy of the full-length peptides in inhibiting *Xanthomonas campestris* pv. *campestris*, as well as that of the short versions of peptide 4 and the C-terminal-modified analogs (r version), tested at (**A**) 15 µM and (**B**) 3 µM. Bars indicate standard errors. Three replicates were performed for each peptide concentration. Two independent experiments were performed. Data were statistically analyzed by a one-way ANOVA followed by the Tukey–Kramer Post Hoc Test. Different letters indicate significant differences at *p* < 0.05.

The sixteen full-length peptides, the six C-terminal-modified, and the three short versions of peptide 4 were tested for their capacity to inhibit Xcc in vitro. Except for peptide 3, which was inactive towards Xcc, all the full-length peptides completely inhibited the growth of the pathogen at 15 µM (Figure 1A).

Among the six C-terminal-modified peptides, five exerted complete inhibition (2r, 4r, 5r, 22r and 24r) and one (6r) was weakly effective at 15 µM (Figure 1A). The short versions of peptide 4 showed a higher variability in inhibiting Xcc, since peptide 4c completely inhibited Xcc, 4c2 had no inhibitory effect and 4c1 was weakly effective. Trichogin GA IV proved inactive against this Gram-negative pathogen (Figure 1A).

Afterward, we reduced the concentration of the peptides to 3 µM in the in vitro assay to identify the most effective peptides with antimicrobial activity. Seven of the full-length peptides (1, 2, 9, 10, 22, 23 and 24) confirmed the complete inhibition observed at 15 µM (Figure 1B). Peptides 6 and 7 showed a partial growth inhibition effect while the remaining peptides (4, 5, 8, 8ApiC, 11 and 25) did not reduce the in vitro Xcc growth. Among the modified versions, at 3 µM, only the peptides 4r, 4c, 5r and 24r confirmed the Xcc growth inhibition detected at 15 µM (Figure 1B).

For the effective peptides, we also determined the MIC and MBC values. Peptides 9, 23, 24 and 24r exerted bacteriostatic activity towards Xcc already at a concentration of 1 µM (Table 2). Peptides 4r, 5r, 10 and 22 showed a MIC of 2 µM whilst peptides 2 and 4c had a MIC of 3 µM (Table 2). Peptides 4r, 5r, 9, 10, 23, 24 and 24r had bactericidal activity at low concentrations ranging from 1 to 3 µM (Table 2). Peptide 3, used as a negative control, showed neither bacteriostatic nor bactericidal activity (Table 2).

### 3.2. Growth Inhibition Activity of Peptide 4r towards Gram-Negative Bacterial Plant Pathogens and the Gram-Positive Bacterium B. subtilis

**Figure 2:**
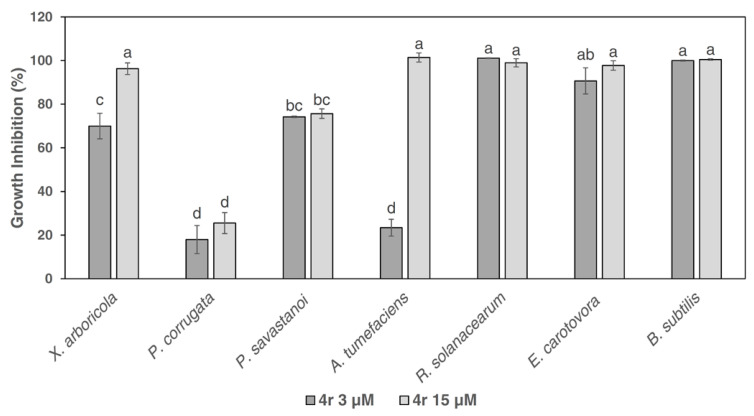
Efficacy of the C-terminal-modified peptide 4r, tested at 15 µM and 3 µM, in inhibiting the in vitro growth of the Gram-negative bacteria *Xanthomonas arboricola*, *Pseudomonas corrugata*, *Pseudomonas savastanoi* pv. *savastanoi*, *Agrobacterium tumefaciens*, *Ralstonia solanacearum*, and *Erwinia carotovora* subsp. *carotovora*, as well as the Gram-positive bacterium *Bacillus subtilis.* Bars indicate standard errors. Three replicates were performed for each bacterial peptide concentration. Two independent experiments were performed. Data were statistically analyzed by a one-way ANOVA followed by the Tukey–Kramer Post Hoc Test. Different letters indicate significant differences at *p* < 0.05.

Given the efficacy of peptide 4r against fungal pathogens [20,21,22] and its bactericidal activity against Xcc, we selected this peptide to test its spectrum of activity against important Gram-negative bacteria and the Gram-positive bacterium *B. subtilis* at the concentrations of 3 µM and 15 µM (Figure 2). *R. solanacearum*, *E. carotovora* subsp. *carotovora* and *B. subtilis* were completely inhibited even at the lowest dose of 3 µM (Figure 2). *X. arboricola* and *A. tumefaciens* were partially inhibited at 3 µM but their growth was completely blocked when the peptide concentration was raised to 15 µM. *P. savastanoi* pv. *savastanoi* was inhibited by approximately 80% at both doses used. *P. corrugata* was the less-sensitive bacterial species, since only about 20% growth inhibition was detected at both concentrations (Figure 2).

### 3.3. Cytotoxicity of Peptide 4r on Tobacco Cells

**Figure 3:**
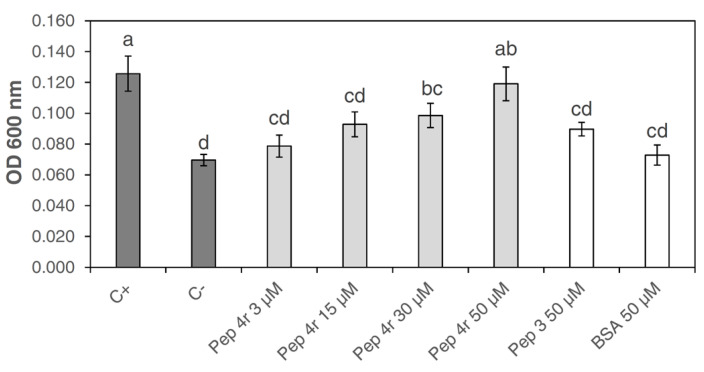
Cytotoxicity analysis evaluated by Evans Blue staining towards tobacco cells treated with peptide 4r at different concentrations. Peptide 3 at 50 µM was included as a non-active peptide because it has no antibacterial activity. Boiled tobacco cells were used as a positive control (C+). Tobacco cells treated with water (C-) or with BSA at 50 µM were used as negative controls. Three technical and three biological replicates were used in three different experiments. Data were statistically analyzed by a one-way ANOVA followed by the Tukey–Kramer Post Hoc Test. Different letters indicate significant differences at *p* < 0.05.

The cytotoxicity was evaluated by estimating the death level of a tobacco cell suspension after treatment with the peptide 4r at different concentrations (3, 15, 30 and 50 µM). As negative controls, the cells were treated with water or BSA. Peptide 3 was included in the experiment for comparative purposes because, as above reported, it did not show any antibacterial activity. As a positive control, the cells were killed by heating in a boiling bath. According to the Evans Blue assay, the cytotoxicity level was determined spectrophotometrically by measuring the amount of the dye released after washing with a methanol solution. The assay showed that the peptide 4r was not cytotoxic at the concentrations of 3 and 15 µM, since comparable absorbance levels were detected with the negative controls (Figure 3). At 30 µM, the peptide 4r was weakly toxic to tobacco cells, while at 50 µM, the peptide showed a cytotoxic effect, similar to the one determined with the positive control (heat-killed cells; Figure 3).

### 3.4. Effect of Peptide Treatment on Cauliflower Plants against Xcc Infection

The efficacy of peptide 4r in reducing Xcc disease symptoms on cauliflower leaves was evaluated both as pre- and post-inoculation treatments. In the pre-inoculation treatment, leaves sprayed with the peptide 4r were allowed to dry and then were inoculated with an Xcc suspension. In the post-inoculation treatment, the peptide was administered 24h post Xcc inoculation. To improve the adhesion to the leaf surface, in all the experiments, the peptide was mixed with the sticker adjuvant pinolene. Treatments with pinolene or water alone were also included as controls.

**Figure 4:**
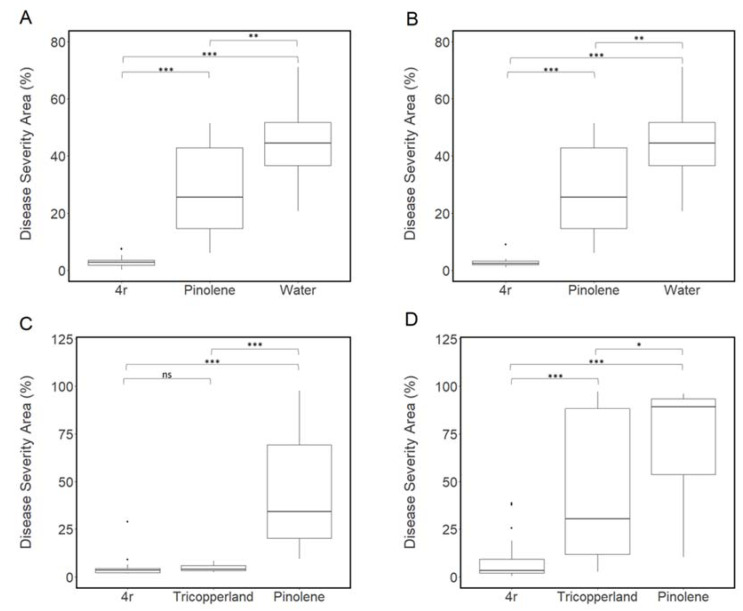
Boxplot of the Disease Severity Area (DSA) caused by *Xanthomonas campestris* pv. *campestris* (Xcc) inoculation on cauliflower cv. “Palla di neve” leaves, subjected to pre- (**A** and **C**; Xcc was inoculated after peptide solution had dried) or post-inoculation (**B** and **D**; peptide solution sprayed 24 h post Xcc inoculation) treatments with peptide 4r, supplemented with the adjuvant pinolene, in comparison to compositions with pinolene alone or with a tribasic copper sulfate formulation (Tricopperland; **C** and **D**). For each treatment, 2 leaves of 3 plants were treated and inoculated. Data from three independent experiments were analyzed together. Asterisks indicate significant differences between the averages of the compared treatments based on a one-way ANOVA followed by Tukey’s honestly significant differences, “***” for *p* < 0.001, “**” for *p* < 0.01, and “*” for *p* < 0.05; ns = not significant. Actual values are plotted on boxplots; boxes range from the first quartile (Q1) to the third quartile (Q3), and the line across the box represents the median. Whiskers extend from Q3—1.5 (Q3−Q1) to Q3 + 1.5 (Q3−Q1). Outliers are represented as black dots.

Results showed that, in both pre- and post-inoculation applications, the pinolene suspension significantly reduced the Disease Severity Area (DSA) as compared with the water treatment (Figure 4A,B). However, the treatment with peptide 4r supplemented with pinolene markedly reduced the DSA value compared to pinolene (Figure 4A,B).

In successive experiments, we compared the efficacy of the peptide 4r supplemented with pinolene with a composition supplemented by a cupric bactericide (tribasic copper sulfate). In the pre-inoculation treatments, both peptide 4r and copper similarly reduced the DSA by about 90% as compared to control plants treated with pinolene alone (Figure 4C). The peptide 4r was also effective as a post-inoculation treatment, with an 87% reduction of DSA compared to control plants treated with pinolene alone (Figure 4D). Interestingly, the post-inoculation peptide treatment was more effective than the copper formulation since, in these plants, the DSA was reduced by only about 36% as compared to control plants treated with pinolene alone (Figure 4D). Leaves treated with water or pinolene always showed visible Xcc symptoms (Supplementary Figure S1). No phytotoxic effect was observed in plants sprayed with peptide 4r or copper (Supplementary Figure S1).

## 4. Discussion

Previously, we have reported that peptides derived by specific amino acid substitutions of the native trichogin GA IV have antifungal activity [20,21,22]. Here, we proved that some of those peptides, as well as de novo synthesized ones, are active at low µM concentrations against the Gram-negative phytopathogenic bacterium Xcc. Trichogin GA IV is not active against this bacterium, confirming the restricted inhibitory activity of natural peptaibols, whose activity at µM levels was demonstrated only against some Gram-positive bacteria [23,31]. However, recent research demonstrated that also the natural trichokonins peptaibols are effective against the Gram-negative rice pathogen *Xanthomonas oryzae* pv. *oryzae* [32].

Only four out of the twenty-give trichogin GA IV-derived peptides tested against Xcc were inactive (peptides 3 and 4c2) or poorly active (peptides 4c1 and 6r) when assayed at 15 µM, which was the highest concentration used in the in vitro experiments. The peptides 4r, 5r, 9, 10, 23, 24, and 24r proved to be the most effective against Xcc, since they displayed MIC and MBC values at the lowest concentrations of 1–3 µM. Their inhibition efficacy seems to not be related to the peptide length, the number and position of lysine in the peptide sequence, or the presence of a C-terminal amide. In the most active peptides, the number of amino acids is 10, 11, or 21, the lysines total 2, 3, 4 or 8; these can be paired or scattered along the molecule and the C-terminal amide is present or not. In-depth structural studies by circular dichroism and 2-dimensional nuclear magnetic resonance, carried out also on some of the peptides herein studied, highlighted the presence of a well-developed helical conformation for trichogin GA IV and its Lys-containing analogs [19,20,22]. In addition, previously reported leakage experiments demonstrated the ability of trichogin and its analogs to modify membrane permeability and cause the release of an entrapped fluorescent dye from small unilamellar lipid vesicles. Those experiments have been performed also on reconstituted membranes, made of lipids extracted from bacteria [19]. Considering the results reported in the literature, we can safely consider the peptide analogs herein studied as being able to effectively interact with bacterial membranes. This ability probably contributes significantly to the observed antimicrobial activity of the peptides at the lowest concentrations tested.

Peptide 4r has previously been shown to be one of the most effective peptides against fungi [20,21,22] and can, therefore, be considered a cross-active peptide. Some peptides appear equally active against Xcc and fungi, while others are more selective. For example, peptides 1, 2, 6 and 7 completely inhibit Xcc at 15 µM, while they are poorly effective towards *B. cinerea* [20], and peptide 6 (full inhibition against Xcc) exerts a strain-dependent inhibition of *P. oryzae* [22]. Current knowledge does not allow us to understand the reasons for the selective efficacy of these peptides in bacteria compared to fungi. Currently, the only study that has investigated the alterations of the cellular activities caused by the synthetic analog of trichogin GA IV peptide 4r in the *P. oryzae* transcriptome highlighted the differential expression of genes involved in the response to oxidative stress, detoxification, autophagic cell death, cell wall biogenesis, degradation and remodeling, melanin and fatty acid biosynthesis, and ion efflux transporters [22]. These are hallmarks of general toxicity, but a more specific effect of the peptides cannot be excluded. Transcriptomic studies would be needed to investigate which processes are impaired in bacterial cells.

The peptide 4r was designated, for its wide range of activity, as a reference peptide for performing in vitro growth inhibition experiments against other Gram-negative pathogenic bacteria, namely, *X. arboricola*, *P. corrugata*, *P. savastanoi* pv. *savastanoi*, *A. tumefaciens*, *R. solanacearum,* and *E. carotovora* subsp. *carotovora*, as well as the Gram-positive bacterium *B. subtilis.* At the concentration of 15 µM, peptide 4r completely inhibited all the tested bacteria except *P. savastanoi* pv. *savastanoi* and *P. corrugata*. In addition, increasing the peptide concentration from 3 to 15 µM did not cause an increase in inhibition against these two *Pseudomonads*. Phytopathogenic *Pseudomonas* species are well-known producers of alginate, forming an exopolysaccharide biofilm [33,34]. The biofilm of *P. aeruginosa* protects the bacterial cells from antimicrobial treatments [35]. Similarly, the lower susceptibility of *P. savastanoi* pv. *savastanoi* and *P. corrugata* to peptide 4r could be attributed to the capacity of these bacteria to produce a biofilm that protects the cells. The inhibition of the BCA bacterium *B. subtilis* by peptide 4r is of particular importance, because it suggests that the amino acid substitutions in the trichogin GA IV sequence determine the toxicity against this Gram-positive bacterium [23]. The sensitivity of *B. subtilis* may preclude the possibility of applying the peptide and the antagonist together.

Our results highlight the superior activity of modified AMPs compared to the native molecules. The Lys insertion is responsible for a stronger and larger spectrum of antibacterial activity, and this is a shared feature of other linear synthetic peptides. In this regard, a library composed of short linear synthetic peptides was evaluated for activity against the phytopathogenic bacteria [36,37]; most of them showed a MIC < 7.5 μM that is close to the MIC values detected in this work. Antimicrobial activity towards plant-pathogenic Gram-negative bacteria (most of the peptides exhibited antibacterial activity at MIC < 12.5 μM) and fungi (MICs ranging between 3 and 100 μM) was also observed for short lipopeptides obtained by chemical synthesis [38]. Recently, synthetic peptides showed bactericidal activity against some strains of *Xylella fastidiosa* [39]. The Lys-containing peptaibols herein described comprise the strong antimicrobial activity of cationic peptides, with a remarkable proteolytic stability given by the presence of the non-coded Aib residues, which are also responsible for their stable and well-defined helical conformations, known to promote peptide–membrane interactions.

An important aspect related to the adoption of AMPs as a tool for plant disease control is phytotoxicity. Baccelli et al. (2022) [21] did not detect any production of reactive oxygen species (ROS) by tomato and *Arabidopsis* leaves sprayed with the peptide 4r at 50 µM. It is noteworthy that ROS are involved in cell signaling but are also considered sensitive indicators of cell damage [40]. We tested the peptide 4r for its cytotoxicity toward tobacco cell cultures. Peptide 4r was not cytotoxic up to a concentration of 15 µM, but it started to kill the plant cells at 30 µM and became highly cytotoxic at 50 µM, a concentration, however, 25 times higher than the MIC value. Likely, the plant barrier represented by the plant cuticle hampers the toxic effect on plant cells. Therefore, peptide 4r can be considered a good candidate for the management of pathogenic bacteria in agriculture.

The analysis of symptoms on white cauliflower leaves sprayed with peptide 4r and inoculated with Xcc highlighted the efficacy of the peptide both as a pre- and post-infection treatment. The efficacy of peptide 4r was similar to that of tribasic copper sulfate (Tricopperland) when applied as a pre-inoculation. Interestingly, plants sprayed with peptide 4r showed lower DSA values than plants sprayed with the cupric fungicide in post-inoculation experiments, which is in agreement with the protectant and not the curative mode of action of copper. These results are encouraging for the future development of biorational compounds with antibacterial activity against Xcc as effective as copper-based products.

The scientific community is now focusing on the analysis of microbial communities perturbed by Xcc infection, in order to isolate indigenous BCAs able to antagonize Xcc [41]. An alternative is the use of secondary metabolites produced by BCAs as molecules that interfere with essential functions of the pathogen such as biofilm formation [42]. In addition, unraveling plant immunity defense components involved in resistance responses to Xcc [43] is fundamental for achieving an integrated approach to the management of this pathogen. Future research should also evaluate the efficacy of these peptide analogs against Xcc in open-field conditions.

In conclusion, we successfully increased the spectrum of activity of trichogin GA IV against Gram-negative bacteria. Indeed, we found several trichogin GA IV peptide analogs with antibacterial activity against important Gram-negative pathogenic bacteria. Moreover, the in vivo effectiveness of peptide 4r may have a positive impact on the management of diseases caused by *X. campestris* pv. *campestris*.

## 5. Patents

The Patents EU 22160196.6 (7/09/22) and US 2022/0330548 A1 (20/10/22) resulted from the work reported in this manuscript.

## Figures and Tables

**Table 1:** Trichogin GA IV analogs used in this work.

Peptide	Sequence	Synthesis
Trichogin GA IV	nOct-Aib-Gly-Leu-Aib-Gly-Gly-Leu-Aib-Gly-Ile-Lol	
1	nOct-Aib-**Lys**-Leu-Aib-**Lys**-Gly-Leu-Aib-Gly-Ile-Lol	[20]
2	nOct-Aib-**Lys**-Leu-Aib-Gly-Gly-Leu-Aib-Gly-Ile-Lol	[20]
2r	nOct-Aib-**Lys**-Leu-Aib-Gly-Gly-Leu-Aib-Gly-Ile-**Leu-NH_2_**	[22]
3	nOct-Aib-Gly-Leu-Aib-**Lys**-Gly-Leu-Aib-Gly-Ile-Lol	[20]
4	nOct-Aib-Gly-Leu-Aib-**Lys-Lys**-Leu-Aib-Gly-Ile-Lol	[20]
4r	nOct-Aib-Gly-Leu-Aib-**Lys-Lys**-Leu-Aib-Gly-Ile-**Leu-NH_2_**	[20]
4c	nOct-Aib-**Lys-Lys**-Leu-Aib-Gly-Ile-Lol	[20]
4c1	nOct-Aib-Gly-Leu-Aib-**Lys-Lys**-Leu-**Leu-NH_2_**	[20]
4c2	nOct-Aib-**Lys-Lys**-Leu-Aib-Gly-Ile-**Leu-NH_2_**	[20]
5	nOct-Aib-**Lys**-Leu-Aib-**Lys**-Gly-Leu-Aib-**Lys**-Ile-Lol	[22]
5r	nOct-Aib-**Lys**-Leu-Aib-**Lys**-Gly-Leu-Aib-**Lys**-Ile-**Leu-NH_2_**	This work
6	nOct-Aib-Gly-Leu-Aib-**Lys-Aib**-Leu-Aib-Gly-Ile-Lol	[20]
6r	nOct-Aib-Gly-Leu-Aib-**Lys-Aib**-Leu-Aib-Gly-Ile-**Leu-NH_2_**	[21]
7	nOct-Aib-**Lys**-Leu-Aib-Gly-**Lys**-Leu-Aib-Gly-Ile-Lol	[20]
8	nOct-Aib-Gly-Leu-Aib-Gly-Gly-Leu-**Api**-Gly-Ile-Lol	[20]
8ApiC	nOct-Aib-Gly-Leu-Aib-Gly-Gly-Leu-**Api**-Ile-Lol	[22]
9	N3Ac-Aib-**Lys**-Leu-Aib-**Lys-Lys**-Leu-Aib-**Lys**-Ile-Lol	This work
10	nOct-Aib-**Lys**-Leu-Aib-**Lys-Lys**-Leu-Aib-**Lys**-Ile-Leu-Aib-**Lys**-Leu-Aib-**Lys-Lys**-Leu-Aib-**Lys**-Ile-Lol	This work
11	nOct-**Toac**-Gly-Leu-Aib-Gly-Gly-Leu-Aib-**Arg**-Ile-Lol	This work
22	nOct-Aib-Gly-Leu-Aib-Gly-**Lys**-Leu-Aib-Gly-Ile-Lol	[22]
22r	nOct-Aib-Gly-Leu-Aib-Gly-**Lys**-Leu-Aib-Gly-Ile-**Leu-NH_2_**	[22]
23	nOct-Aib-**Lys**-Leu-Aib-**Lys-Lys**-Leu-Aib-Lys-Ile-Lol	This work
24	nOct-Aib-**Lys**-Leu-Aib-**Lys-Lys**-Leu-Aib-Gly-Ile-Lol	This work
24r	nOct-Aib-**Lys**-Leu-Aib-**Lys-Lys**-Leu-Aib-Gly-Ile-**Leu-NH_2_**	This work
25	nOct-Aib-Gly-Leu-Aib-Gly-Gly-Leu-Aib-**Lys**-Ile-Lol	[21]

Amino acid substitutions compared to the native sequence are highlighted in bold. Lol: leucinol; Aib: α-aminoisobutyric acid; Api: indomethacin; Toac: 2,2,6,6-tetramethyl-N-oxyl-4-amino-4-carboxylic acid.

**Table 2:** Minimal Inhibitory Concentration (MIC) and Minimal Bactericidal Concentration (MBC) of the most effective trichogin GA IV analogs.

Peptides	0.5 µM	1 µM	2 µM	3 µM
2	-	-	-	MIC
3	-	-	-	-
4r	-	-	MIC	MBC
4c	-	-	-	MIC
5r	-	-	MIC and MBC	
9	-	MIC	MBC	
10	-	-	MIC and MBC	
22	-	-	MIC	-
23	-	MIC and MBC		
24	-	MIC	MBC	
24r	-	MIC	MBC	

Dashes (-) indicate the absence of bacteriostatic or bactericidal activity. MIC and MBC were determined at decreasing concentrations (3, 2, 1 and 0.5 µM).

## Data Availability

All data are available from the corresponding author upon request.

## Supplementary Materials

The following supporting information can be downloaded at: https://www.mdpi.com/article/10.3390/microorganisms11020480/s1, Figure S1: Disease symptoms caused by *Xanthomonas campestris* pv. *campestris* (Xcc) on cauliflower cv. “Palla di neve” leaves subjected to treatments with peptide 4r supplemented with pinolene (adjuvant) in comparison with tribasic copper sulfate (Tricopperland), pinolene or water treatments.
