# Supplementary material for: Efficacy of Trichoderma longibrachiatum Trichogin GA IV Peptaibol analogs against the Black Rot Pathogen Xanthomonas campestris pv. campestris and other Phytopathogenic Bacteria
Source: Microorganisms. 2023 Feb 14;11(2):480. doi: 10.3390/microorganisms11020480 (PMC9967956; doi:10.3390/microorganisms11020480)
Supplement: Supplementary file 1 [file microorganisms-11-00480-s001.zip › microorganisms-2200770-supplementary.pdf]

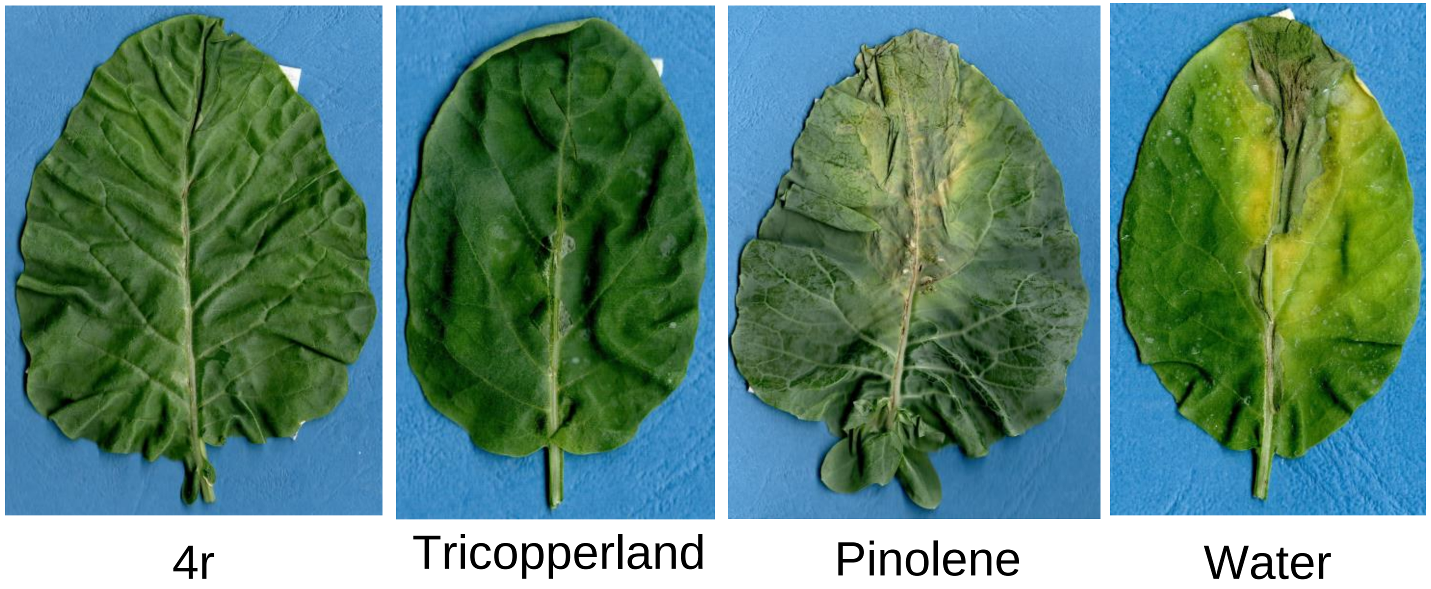

Figure S1. Disease symptoms caused by Xanthomonas campestris pv. campestris (Xcc) on cauliflower cv. “Palla di neve” leaves subjected to treatments with peptide 4r supplemented with pinolene (adjuvant) in comparison with tribasic copper sulfate (Tricopperland), pinolene or water treatments.
